# Bridging Diagnostic Gaps: Utilising HiCrome Agar and Tetrazolium Reduction Medium for the Rapid and Presumptive Identification and Speciation of Candida Species in Vulvovaginal Candidiasis in Low-Resource Environments

**DOI:** 10.7759/cureus.65601

**Published:** 2024-07-28

**Authors:** Lavanya Balaji, Jayakumar Subramaniam

**Affiliations:** 1 Department of Microbiology, Saveetha Medical College and Hospitals, Saveetha Institute of Medical and Technical Sciences, Saveetha University, Chennai, IND

**Keywords:** candida species infection, chromogenic agar, tetrazolium reduction medium, hicrome agar, recurrent vulvovaginal candidiasis

## Abstract

Background

Vulvovaginal candidiasis (VVC) is a common fungal infection caused by an overgrowth of *Candida *species, primarily *Candida* *albicans* (*C. albicans*). Using HiCrome agar and tetrazolium reduction medium offers cost-effectiveness in *Candida *detection by eliminating the need for additional tests, reducing equipment costs compared to automated systems, and simplifying workflow with direct species identification while maintaining high specificity. They expedite detection by directly identifying *Candida* species based on colony colour, bypassing the multiple steps of phenotypic methods. This efficiency saves time in the laboratory, providing rapid results without the extended processing times associated with automated systems and facilitating prompt diagnosis and treatment decisions. These diagnostic tools are especially valuable in low-resource environments where a quick and accurate diagnosis of VVC is crucial for effective treatment and management of antifungal resistance.

Aims and objectives

This study aims to evaluate the efficacy of HiCrome agar and tetrazolium reduction medium's efficacy in speciating* Candida* species in VVC cases.

Materials and methods

A cross-sectional observational study was conducted at Saveetha Medical College and Hospitals, Chennai, India, over six months. High vaginal swabs from 126 patients suspected of VVC were collected and plated on Sabouraud dextrose agar (SDA), HiCrome Candida differential agar (Himedia, Mumbai, India), and tetrazolium reduction medium. The results were compared with those obtained from the VITEK2 compact system (bioMérieux, Marcy-l'Étoile, France).

Results

Of the 126 samples, 74.6% showed single yeast infections, 7.9% displayed mixed yeast infections, and 17.5% showed no growth. A total of 114 *Candida* isolates were identified. Both HiCrome agar and tetrazolium reduction medium accurately identified all isolates, with complete concordance with the VITEK2 compact system. The most commonly isolated species were *C. albicans* (55.2%), *Candida tropicalis* (32.4%), *Candida glabrata *(8.8%), and *Candida parapsilosis* (3.6%). Both media provided rapid and accurate presumptive identification in low-resource settings.

Conclusions

HiCrome agar and tetrazolium reduction medium demonstrated high sensitivity and specificity in identifying Candida species. These methods are reliable for rapid and accurate diagnosis, particularly in resource-limited settings. However, they may require supplementary tests for definitive species identification. The adoption of these diagnostic tools represents a significant advancement in clinical microbiology, improving VVC management and addressing antifungal resistance.

## Introduction

Vulvovaginal candidiasis (VVC), commonly referred to as a yeast infection, is a widespread fungal infection impacting the vulva and vagina. It is predominantly caused by an overgrowth of *Candida* species, mainly *Candida* *albicans*, a type of yeast normally present in small quantities in the vagina, mouth, digestive tract, and on the skin [1]. Disruption of the microbial balance in the vagina allows *Candida* to multiply excessively, leading to infection. This condition affects millions of women worldwide, causing significant discomfort and disruption to daily life [2]. *Candida *species typically coexist with protective bacteria in the vagina, maintaining an acidic environment that prevents the overgrowth of pathogens. Factors such as uncontrolled diabetes, antibiotic use, hormonal changes, immunosuppression, and diet and lifestyle choices can disrupt this delicate balance, increasing susceptibility to VVC [3]. Antibiotics can reduce the population of protective bacteria, while hormonal fluctuations, immunosuppression, and poor diet or hygiene can also contribute to yeast overgrowth [4]. When *Candida* proliferates, it adheres to vaginal epithelial cells, forming biofilms that enhance its survival and resistance to treatment. This overgrowth triggers an inflammatory response, causing the characteristic symptoms of VVC [5]. Symptoms may vary in intensity and encompass strong itching and discomfort of the vulva and vaginal area, a dense, white, cottage cheese-like discharge without an unpleasant smell, inflammation and enlargement of the vulva and vaginal tissues, discomfort during sexual intercourse and urination, and a sensation of burning in the affected region [6]. Diagnosing VVC involves a comprehensive approach that includes clinical assessment and laboratory tests. A typical diagnostic protocol includes a clinical examination to evaluate symptoms and perform a physical assessment of the vulva and vagina. Microscopy is used to examine a sample of vaginal discharge to detect *Candida* cells and pseudo hyphae indicative of a yeast infection. Vaginal cultures are also used to grow and identify *Candida* species, although this is not always necessary for uncomplicated cases. Additionally, vaginal pH testing, which is usually below 4.5 in VVC, helps differentiate it from conditions like bacterial vaginosis, which is characterised by elevated pH levels. Managing recurrent or complicated cases may require additional testing to identify underlying conditions or resistant *Candida* species [7-9]. Treatment for VVC focuses on eradicating the infection, alleviating symptoms, and preventing recurrence. Antifungal medications are the primary treatment option and include topical antifungals such as over-the-counter and prescription creams, ointments, suppositories, and tablets containing agents like clotrimazole, miconazole, and tioconazole, which are applied directly to the affected area. Oral antifungals like fluconazole are used for severe or recurrent infections, with a single dose often being effective, though chronic cases may require longer courses [10]. VVC is the most common yeast infection in humans, affecting about 75% of women at least once in their lifetime. Recurrent VVC (RVVC) occurs when a person has more than three episodes in a year and affects almost 8% of women globally. It often needs ongoing antifungal treatment with azole drugs to reduce the chance of the infection coming back [11]. The persistent effectiveness of azoles and insufficient immune-mediated clearance are important contributors to the reoccurrence of the disease [12].

Identifying the specific *Candida* species responsible for the infection is crucial due to variations in their susceptibility to antifungal medications. For example, *Candida glabrata *(*C. glabrata*) often shows resistance to azoles, necessitating higher doses or alternative antifungal agents like echinocandins [13]. Similarly, *Candida krusei *(*C. krusei*) is inherently resistant to fluconazole, requiring alternative treatments. Accurate speciation enables targeted therapy, ensuring the most effective antifungal agent is selected, minimising the risk of treatment failure. In recurrent or chronic VVC, speciation is even more important because non-*albicans* *Candida* (NAC) species, which are often more resistant to standard treatments, are frequently involved. Identifying the specific species helps tailor treatment regimens and allows for early intervention in cases where resistance to standard treatments is detected [14]. Detecting mixed infections with multiple yeast species in clinical samples is crucial for effective patient treatment. The challenge of quickly identifying these mixed cultures on traditional Sabouraud’s dextrose agar (SDA) has prompted the development of chromogenic media. This advancement not only helps in the rapid identification of mixed yeast infections from clinical samples but also provides results 24-48 hours earlier compared to standard identification methods. HiCrome agar and tetrazolium reduction medium are valuable tools in the laboratory for the presumptive speciation of *Candida* in mixed yeast infections [15]. These media enable the differentiation and presumptive identification of *Candida* species based on their unique biochemical properties and colony morphology. HiCrome agar contains substrates that react with specific enzymes produced by different *Candida* species, resulting in colonies displaying distinct colours [16]. Tetrazolium reduction agar differentiates *Candida* species based on their ability to reduce tetrazolium salt into insoluble coloured formazans resulting in colour change [17]. Both media provide a relatively quick, visual, cost-effective, and accurate method for identifying *Candida* species, aiding in appropriate treatment selection and improving patient outcomes in antifungal resistance cases. Despite some limitations, these methods are invaluable in the clinical microbiology laboratory for managing *Candida*-related infections. Therefore, our study aims to evaluate the effectiveness and utility of HiCrome agar and tetrazolium reduction medium in the speciation of *Candida* species in cases of VVC.

## Materials and methods

Study design and setting

This cross-sectional observational study was conducted at the Central Laboratory, Department of Microbiology, Saveetha Medical College and Hospitals, Chennai, India. The study was carried out over six months, from December 2023 to May 2024, after obtaining ethical clearance (258/07/2023/PG/SRB/SMCH). High vaginal swabs were collected from 126 patients suspected of VVC.

Inclusion criteria 

Women diagnosed with VVC, based on clinical symptoms and the presence of vaginal discharge, were included in the study.

Exclusion criteria 

Women with co-infections (e.g., bacterial vaginosis, trichomoniasis), pregnant women, and those who have used antifungal treatments within the last month were excluded from the study.

The samples were then directly plated on SDA, HiCrome Candida differential agar (Himedia, Mumbai, India), and tetrazolium reduction agar, and incubated at 37°C for 48 hours aerobically.

HiCrome Candida differential agar

The HiCrome Candida differential agar is a selective and differential chromogenic medium designed to distinguish between *Candida* species. Inoculate the clinical sample onto the HiCrome Candida differential agar and incubate at 37°C for 24-48 hours aerobically. Using specific enzymatic activities and various chemical substrates, the medium causes *Candida* colonies to display different colours, allowing for presumptive identification on the isolation plate, as referenced in Table 1.

**Table 1:** Identification of Candida species based on colour production by HiCrome agar.

Organism	Colour produced on HiCrome agar
Candida albicans	Light green
Candida tropicalis	Steel blue to purple
Candida glabrata	Cream to white
Candida parapsilosis	White to light pink
Candida krusei	Purple, fuzzy

Tetrazolium reduction medium

The tetrazolium reduction medium is utilised for distinguishing between different *Candida *species based on the colours they produce. Various species of *Candida *reduce tetrazolium to varying extents. To prepare the medium, dissolve 1 gm of peptone, 4 gm of glucose, and 0.1 gm of beef extract in 100 mL of distilled water and adjust the pH to between 5.6 and 6.2. After autoclaving at 121°C for 15 minutes, cool to a temperature that can be touched by hand and add 20 mg of tetrazolium and 50 mg of neomycin. Inoculate the clinical sample onto the tetrazolium reduction medium and incubate at 37°C for 24-48 hours aerobically. After incubation, the resulting colours are observed for presumptive identification [18], as shown in Table 2.

**Table 2:** Identification of Candida species based on colour production by TRM. TRM: Tetrazolium reduction medium

Organism	Colour produced on TRM
Candida albicans	Light pink
Candida tropicalis	Dark, maroon-red
Candida glabrata	Pale pink
Candida parapsilosis	Rose pink
Candida krusei	Pink and dry

Clinical samples with single yeast infections on HiCrome agar and tetrazolium reduction medium were directly identified using the VITEK2 system (bioMérieux, Marcy-l'Étoile, France) from the SDA. Samples showing mixed yeast infections with multiple *Candida* species on HiCrome agar and tetrazolium reduction medium were cultured again on SDA and incubated aerobically at 37°C for 24-48 hours. Pure colonies from these cultures were then identified and confirmed using the VITEK2 system. This method ensured precise identification of both single and mixed *Candida* infections, utilising the specific screening capability of chromogenic agars and the reliable species identification provided by the VITEK2 system.

Identification by VITEK2 system

For identification using the VITEK2 system, a pure culture of the organism was suspended in 3.0 mL of sterile saline (0.45% NaCl, pH 4.5 to 7.0) in a polystyrene test tube. Turbidity was adjusted to a McFarland 2.0 standard and measured using a DensiCHEK turbidity meter (bioMérieux, Marcy-l'Étoile, France). The culture suspension was automatically dispensed into test cards, which were sealed and placed in the VITEK2 instrument for incubation at 35.5°C for 18 hours. Optical density readings were automatically recorded every 15 minutes. The final identification results were compared against a database for accurate species identification. The evaluation included the following control strains: *C. albicans* ATCC 90028 and *Candida tropicalis *(*C. tropicalis*) ATCC 750.

The results from HiCrome Candida differential agar and tetrazolium reduction medium were tabulated and compared with the automated VITEK2 system to assess their effectiveness in the presumptive identification of *Candida* species. The data was compiled into a master chart using Microsoft Excel (Microsoft® Corp., Redmond, WA, USA), and the correlation between the media was analysed using a Chi-square test (χ² test). A p-value below 0.05 indicated statistical significance.

## Results

In this study, we employed both HiCrome agar and tetrazolium reduction medium along with SDA as primary plating media for high vaginal swab samples (n = 126). Single yeast infections were observed in 74.6% of the isolates, while mixed yeast infections were identified in 7.9% of the samples. Approximately 17.5% of the samples showed no growth. A total of 114 *Candida *isolates were identified from the 126 samples. Using the VITEK2 compact system to identify *Candida* isolates, the distribution was as follows: *C. albicans* was the most commonly isolated species in single yeast infections (n = 53), followed by *C. tropicalis *(n = 31), *C. glabrata* (n = 6), and* Candida parapsilosis* (*C. parapsilosis*) (n = 4). In cases of mixed yeast infections, combinations of *C. albicans* and *C. tropicalis* (n = 6) and *C. albicans* and *C. glabrata *(n = 4) were detected.

**Figure 1:**
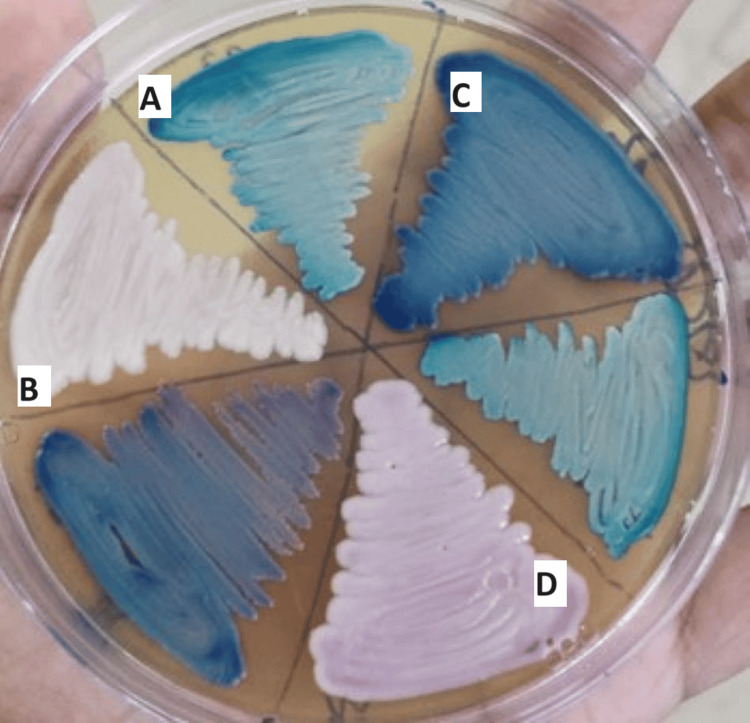
Presumptive identification of Candida isolates based on colour production by HiCrome agar. A) Green-coloured colonies indicate Candida albicans; B) White to cream-coloured colonies indicate Candida glabrata; C) Steel blue colonies indicate Candida tropicalis; D) Pink-coloured colonies indicate Candida parapsilosis

SDA showed creamy white to beige colonies that were smooth and pasty, measuring about 1-3 mm in diameter after 24-48 hours at 30-37°C. HiCrome Candida differential agar and tetrazolium reduction medium successfully identified all 114 *Candida* isolates to the species level, with complete concordance with the VITEK2 compact system results. There were no significant differences in growth rate or colony size between HiCrome agar and tetrazolium reduction medium. Both media effectively supported the growth of all isolates under standard conditions (30-37°C for 24-48 hours). The most commonly isolated species were* C.* *albicans* (55.2%),* C. tropicalis* (32.4%), *C. glabrata* (8.8%), and *C. parapsilosis* (3.6%). After 18-24 hours, colonies on HiCrome agar and tetrazolium reduction medium displayed characteristic colours, becoming more pronounced after 48 hours. Green colonies indicated* C. albicans*, steel blue colonies indicated *C. tropicalis*, smooth white colonies indicated *C. glabrata*, and light pink colonies indicated *C. parapsilosis*, matching the manufacturer's instructions (Figure 1).

**Figure 2:**
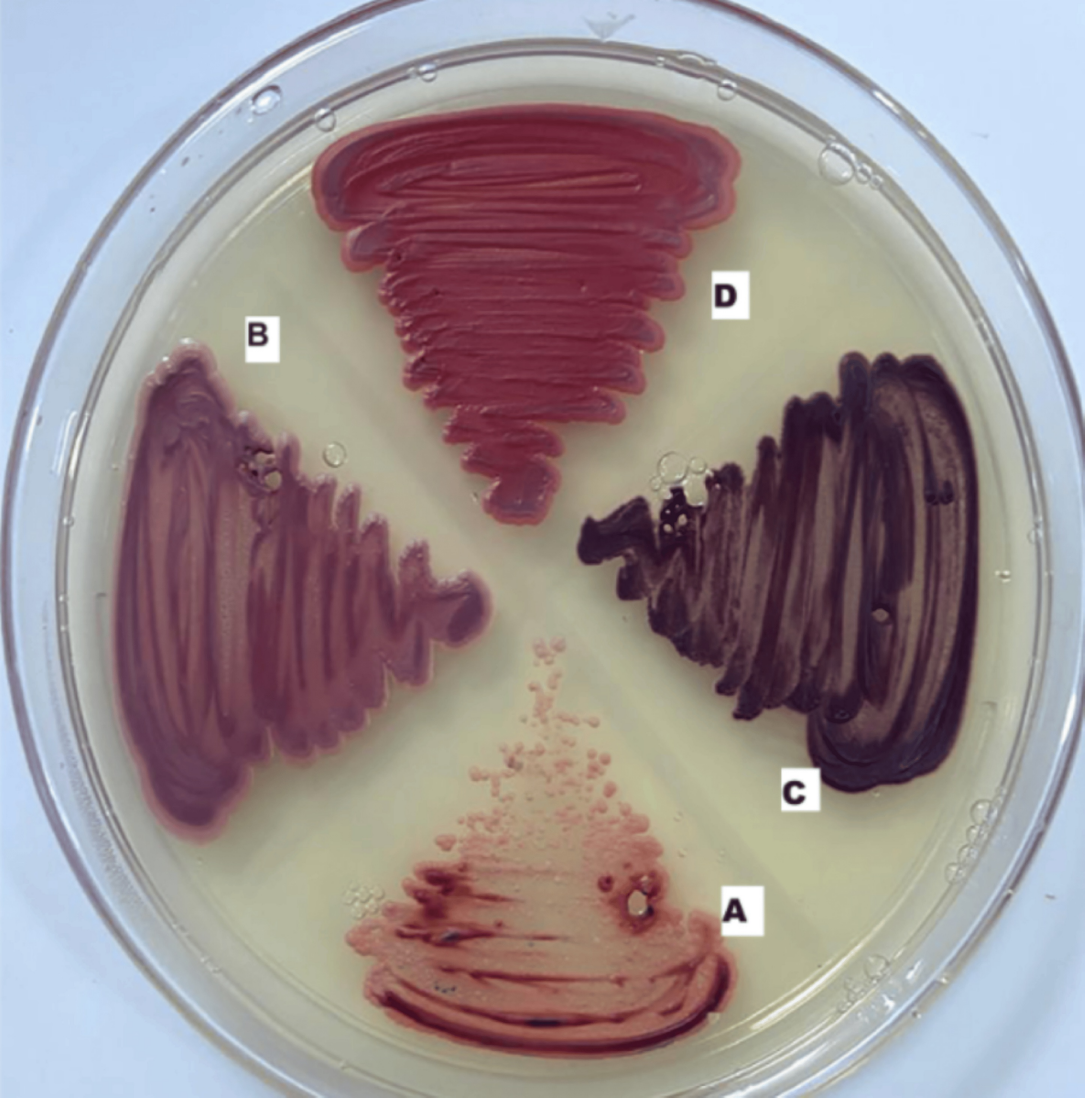
Presumptive identification of Candida isolates by tetrazolium reduction medium. A) Candida albicans: light pink colonies; B) Candida parapsilosis: rose-pink colonies; C) Candida tropicalis: maroon to dark red colonies; D) Candida glabrata: bright pink colonies

The colours differed on the tetrazolium reduction medium: *C. albicans *appeared as light pink colonies, *C. tropicalis* as maroon to dark red colonies, *C. parapsilosis* as rose-pink colonies, and *C. glabrata* as bright pink colonies (Figure 2).

**Figure 3:**
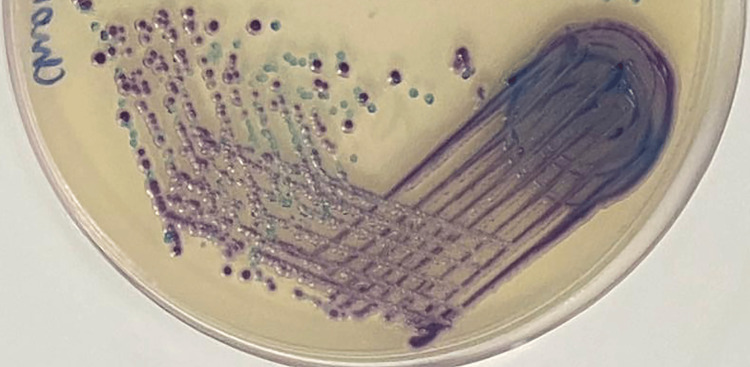
Presumptive identification of two Candida isolates in mixed yeast infections by HiCrome agar. *Candida albicans* is depicted by the production of green-coloured colonies whereas *Candida tropicalis* is depicted by the production of steel blue colonies.

**Figure 4:**
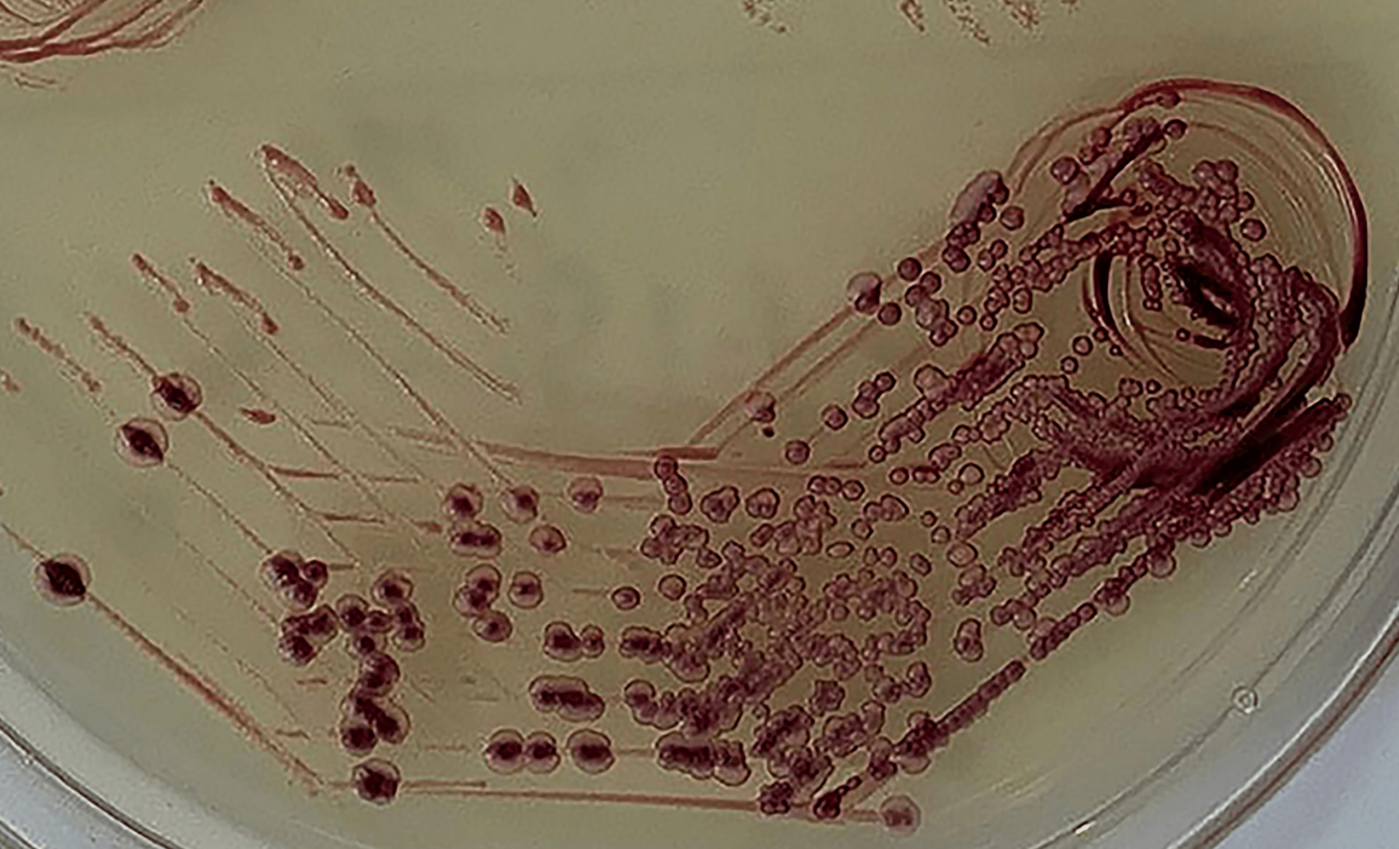
Presumptive identification of two Candida isolates in mixed yeast infections by tetrazolium reduction medium. *Candida albicans* are depicted as light pink colonies, and *Candida tropicalis* are depicted as dark-maroon colonies.

In cases of mixed yeast infections, both HiCrome agar and tetrazolium reduction medium displayed two-coloured colonies, indicating the presence of two different *Candida *species, as shown in Figures 3-4.

In our study comparing the performance of HiCrome agar, tetrazolium reduction medium, and the VITEK2 compact system for the identification and speciation of *Candida* isolates, all three methods demonstrated complete concordance in identifying the four most common *Candida* species: *C. albicans, C. tropicalis, C. parapsilosis, and C. glabrata.* Each method accurately identified 63 isolates of *C. albicans*, 37 isolates of *C. tropicalis*, four isolates of *C. parapsilosis*, and 10 isolates of *C. glabrata*, totalling 114 isolates across all species. Statistical analysis showed no significant differences (p = 1.00) among the three methods, highlighting their comparable performance in clinical microbiology settings. These findings support the reliability and utility of both HiCrome agars and tetrazolium reduction medium as effective tools for the rapid and accurate presumptive identification of *Candida* species, essential for guiding appropriate antifungal therapy in cases of VVC and other *Candida*-related infections. The comparison of the two chromogenic media with the VITEK2 system was evaluated and tabulated, as shown in Table 3.

**Table 3:** The total number of isolates of Candida identified by the VITEK2 compact system and two media. The data are presented as numbers (N). The p-value is calculated using the Chi-square test, with significance considered when the p-value is less than 0.05. In this case, the p-value is 1.00, which is considered not significant. *C. albicans*: *Candida albicans*; *C. tropicalis*: *Candida tropicalis*; *C. parapsilosis*: *Candida parapsilosis*; *C. glabrata*: *Candida glabrata*

	*C. albicans* (n)	*C. tropicalis* (n)	*C. parapsilosis* (n)	*C. glabrata* (n)	Total (n)	p-value
HiCrome agar	63	37	4	10	114	1.00
Tetrazolium reduction medium	63	37	4	10	114	
VITEK2 compact system	63	37	4	10	114	

## Discussion

*C. albicans *remains the predominant species isolated in cases of VVC, consistent with several studies where *C. albicans* accounted for a significant majority of isolates [19-21]. Notably, our study also highlights a substantial prevalence of mixed yeast infections (17%), which is higher than in three other studies, which had prevalence rates of 13%, 5%, and 0.12% [22-24]. Traditionally, laboratories faced challenges in accurately identifying NAC species, relying primarily on the germ tube test for initial identification without subsequent species-level testing or antifungal susceptibility profiling. This approach, prevalent in resource-limited settings, often leads to suboptimal diagnosis and treatment decisions, potentially contributing to the emergence of antifungal resistance [25,26]. In our study, we evaluated the efficacy of HiCrome agar and tetrazolium reduction medium in identifying *Candida *species based on colony morphology and colour differentiation. Both methods demonstrated high sensitivity and specificity, accurately identifying all 114 *Candida *isolates, including mixed infections. This aligns with previous studies by Nadeem et al. [25] and Pravin Charles et al. [27] affirming the reliability of these mediums in clinical settings. While the literature on tetrazolium reduction medium for *Candida* species identification is limited, Giri and Kindo [18] demonstrated its reliability in speciation. Similarly, Denny and Partridge [17] found that the tetrazolium reduction medium is a rapid, fairly accurate, and straightforward method for differentiating *Candida* species from other yeasts in mixed yeast infections, especially in vaginal samples. Our study confirms these findings, as tetrazolium reduction medium successfully speciated all *Candida* isolates, including those from both single and mixed yeast infections.

Chromogenic agars emerged as a pivotal tool in our research, enabling the early identification of *Candida *species within mixed cultures based solely on colony colour. This capability is crucial as conventional methods often fail to detect mixed infections accurately. The research conducted by Willinger and Manafi emphasised the utility of chromogenic agars for isolating and presumptively identifying yeasts, particularly in detecting mixed cultures in clinical samples. They reported high specificity rates: 98.8% for *C. albicans*, 99.8% for* C. tropicalis*, and 100% for *C. krusei.* Our study supports these findings, with our chromogenic media successfully identifying all tested *Candida* species presumptively. This underscores the effectiveness of chromogenic agars in identifying mixed infections, thereby improving treatment approaches and preventing drug resistance [22]. Comparison with the VITEK2 compact system showed no statistical difference in *Candida* identification and speciation between chromogenic mediums and this automated system. This suggests that chromogenic agars can serve as a reliable alternative for presumptive identification and speciation in mixed yeast infections of VVC, particularly in settings where rapid diagnosis is imperative. In clinical practice, swiftly and accurately identifying *Candida* species is crucial for guiding appropriate antifungal therapy. Advanced diagnostic tools like chromogenic agars and tetrazolium reduction medium represent a significant advancement, particularly in regions with limited resources and expertise. Future research to enhance the diagnostic process for identifying *Candida* in low-resource settings using HiCrome agar and tetrazolium reduction medium could focus on several key areas. Incorporating novel biomarkers or genetic markers could lead to more accurate and rapid identification of *Candida *species, reducing false negatives and positives. Optimising the nutrient and chromogenic substrate composition can improve growth and colour differentiation, making it easier to distinguish closely related species. Using multiple chromogens targeting different enzymatic activities and adding selective agents to inhibit non-target organisms can further enhance specificity and reduce misidentification, improving diagnostic accuracy. These methods streamline diagnosis and facilitate targeted treatment approaches, thereby mitigating the risk of antifungal resistance.

Limitations of our study

HiCrome agar aids in distinguishing *Candida* species by colony colour and morphology but may not always provide definitive species identification. Supplementary biochemical or molecular tests are often required for accuracy. Interpretation of colony colours and morphology is subjective, demanding experienced personnel for precision. Variations in colony colour, like different shades of green between closely related species such as *C. albicans* and *Candida dubliniensis* (*C. dubliniensis*), can cause confusion. However, relying solely on colour for identification is insufficient, especially for pathogens like *Candida auris* (*C. auris*),* Candida kefyr*​​​​​​​* *(*C. kefyr*), and*Candida famata*​​​​​​​ (*C. famata*), which lack specific colour markers. Additionally, tetrazolium reduction medium may inaccurately differentiate species, increasing the risk of false results, particularly in cases involving non-*Candida* yeasts in mucosal or skin scrapings [17]. Non-*Candida* yeasts tend to grow poorly on tetrazolium reduction medium, so it is necessary to use phenotypic and automated methods, especially emerging ones, for identification and confirmation. Factors such as pH, temperature, and medium composition can influence the reduction process, potentially leading to inconsistent results. Future research to improve *Candida* species identification should focus on developing chromogenic and tetrazolium reduction media with enhanced specificity and sensitivity. Expanding reference databases with comprehensive genetic and phenotypic profiles of *Candida* species, including rare strains, will improve accuracy.

## Conclusions

Our research underscores the importance of utilising advanced diagnostic tools such as chromogenic agars and tetrazolium reduction medium to accurately identify *Candida* species linked to VVC. These methods have demonstrated exceptional sensitivity and specificity in identifying *C. albicans, C. tropicalis, C. parapsilosis*, and *C. glabrata,* essential for guiding effective antifungal therapy and addressing antimicrobial resistance in mixed yeast infections. However, it's important to recognise the limitations of chromogenic agars, as they may not always provide definitive species identification, necessitating additional testing for confirmation. Despite these challenges, adopting these diagnostic tools represents a notable advancement in clinical microbiology, particularly valuable in resource-limited settings. Future research should continue to explore and refine these diagnostic modalities to further enhance their efficacy and accessibility in clinical practice.
